# Unlocking high-value football fans: unsupervised machine learning for customer segmentation and lifetime value

**DOI:** 10.3389/fspor.2024.1362489

**Published:** 2024-08-22

**Authors:** Karim Chouaten, Cristian Rodriguez Rivero, Frank Nack, Max Reckers

**Affiliations:** ^1^ Faculty of Science, University of Amsterdam, Amsterdam, Netherlands; ^2^ AFC Ajax N.V, Amsterdam, Netherlands; ^3^ Centre for Engineering Research in Intelligent Sensors and Systems (CeRISS), Cardiff Metropolitan University Cardiff, United Kingdom; ^4^ Department of Mining, Industrial and ICT Systems Engineering, Universitat Politècnica de Catalunya Barcelona Tech (UPC), Barcelona, Spain

**Keywords:** football, clustering analysis, artificial intelligence, machine learning, customer segmentation, customer lifetime value, RFM model

## Abstract

**Introduction:**

In the modern competitive landscape of football, clubs are increasingly leveraging data-driven decision-making to strengthen their commercial positions, particularly against rival clubs. The strategic allocation of resources to attract and retain profitable fans who exhibit long-term loyalty is crucial for advancing a club's marketing efforts. While the Recency, Frequency, and Monetary (RFM) customer segmentation technique has seen widespread application in various industries for predicting customer behavior, its adoption within the football industry remains underexplored. This study aims to address this gap by introducing an adjusted RFM approach, enhanced with the Analytic Hierarchy Process (AHP) and unsupervised machine learning, to effectively segment football fans based on Customer Lifetime Value (CLV).

**Methods:**

This research employs a novel weighted RFM method where the significance of each RFM component is quantified using the AHP method. The study utilizes a dataset comprising 500,591 anonymized merchandising transactions from Amsterdamsche Football Club Ajax (AFC Ajax). The derived weights for the RFM variables are 0.409 for Monetary, 0.343 for Frequency, and 0.248 for Recency. These weights are then integrated into a clustering framework using unsupervised machine learning algorithms to segment fans based on their weighted RFM values. The simple weighted sum approach is subsequently applied to estimate the CLV ranking for each fan, enabling the identification of distinct fan segments.

**Results:**

The analysis reveals eight distinct fan clusters, each characterized by unique behaviors and value contributions: The Golden Fans (clusters 1 and 2) exhibit the most favourable scores across the recency, frequency, and monetary metrics, making them relatively the most valuable. They are critical to the club's profitability and should be rewarded through loyalty programs and exclusive services. The Promising segment (cluster 3) shows potential to ascend to Golden Fan status with increased spending. Targeted marketing campaigns and incentives can stimulate this transition. The Needs Attention segment (cluster 4) are formerly loyal fans whose engagement has diminished. Re-engagement strategies are vital to prevent further churn. The New Fans segment (clusters 5 and 6) are fans who have recently transacted and show potential for growth with proper engagement and personalized offerings. Lastly, the Churned/Low Value segment (clusters 7 and 8) are fans who relatively contribute the least and may require price incentives to potentially re-engage, though they hold relatively lower priority compared to other segments.

**Discussion:**

The findings validate the proposed method's utility through its application to AFC Ajax's Customer Relationship Management (CRM) data and provides a robust framework for fan segmentation in the football industry. The approach offers actionable insights that can significantly enhance marketing strategies by identifying and prioritizing high-value segments based on the club's preferences and requirements. By maintaining the loyalty of Golden Fans and nurturing the Promising segment, football clubs can achieve substantial gains in profitability and fan engagement. Additionally, the study underscores the necessity of re-engaging formerly loyal fans and fostering new fans' growth to enable long-term commercial success. This methodology not only aims to bridge a research gap, but also equips marketing practitioners with data-driven tools for effective and efficient customer segmentation in the football industry.

## Graphical Abstract

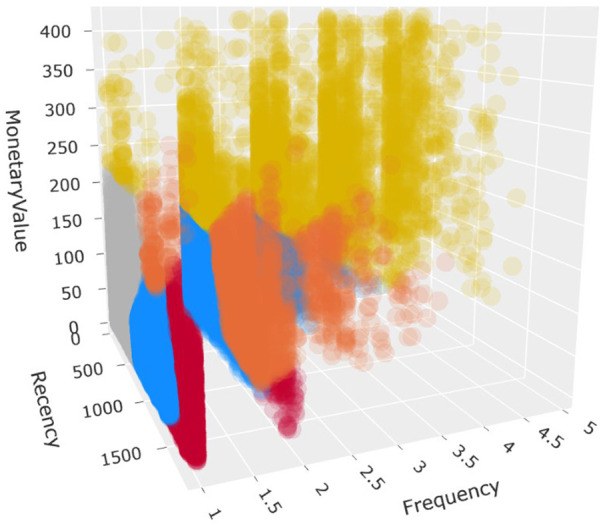

Shows a 3-dimensional scatterplot of the developed fan segments, where observations represent individual football fans. The x-axis includes the fan's Recency, y-axis Monetary, and z-axis Frequency values. The color defines the segment type: Golden Fan (yellow), Promising (orange), Needs Attention (red), New Fan (blue) and Churned/Low Value (gray).

## 1 Introduction

Elite football clubs nowadays are collecting, processing, and analyzing large-scale amounts of player and football related data, in order to improve their competitive position with regard to other rival football clubs (1). One of those football clubs is Amsterdamsche Football Club Ajax (AFC Ajax), which also is the football club that relates to and facilitates this research. According to the multi-year strategic programme of Ajax, the football club wants to structurally compete for the top 20 in the UEFA Champions League. One major contributor to this goal is the digitalization of the organization, and the structural adoption and implementation of data-driven decision making. Another crucial contributor and prerequisite are the structural increase of the so-called operational revenue, which includes all the commercial income excluding all the income generated from transactions of player transfers. The goal hereof is to avoid strong seasonal revenue fluctuations which are vigorously correlated to the football-related performance in matches and competitions.

To advance in achieving this challenging goal, besides the collection of player and football related data, Ajax also has been collecting large amounts of data of its football fans (read: customers) for several years. Most of this data is directly stored in, or linked to, the Customer Relationship Management (CRM) of Ajax. This study therefore aims to fill the business gap between the mentioned goals of Ajax's multi-year strategic programme, by applying unsupervised learning techniques for clustering analysis, in order to effectively and efficiently utilize the collected football fan data stored in the CRM for filling in the currently existing business gap. This in order to derive deeper and actionable insights regarding the range of profitability among football fans, but also to optimize and more accurately offer the appropriate products and services to the befitting audience at the right time. This is then done by using improved communication and (digital) marketing campaigns targeted on specific niches, as each fan has its own and unique connection with the football club. Exploiting the CRM by clustering the football fans into segments may also result in improved effectivity and efficiency of Ajax's resources, as data-driven decision making can be applied for enhancing the performance of its marketing activities, which eventually may lead to an improved match between the supply of Ajax and the demand of the fan. From the academic point of view, there are numerous studies available (2–8) regarding the application of clustering techniques for customer segmentation across various industries, such as banking, retail, e-commerce, marketing, hospitality and healthcare. There, however, is a lack of research regarding this topic for the football industry, and the sports industry as a whole.

The research positioning of this study therefore is to contribute to the existing research gap, by applying clustering techniques for customer segmentation within the football industry. Based on commonly used frameworks for customer segmentation in other industries, the approach of this study is implemented by using Ajax fan data retrieved from the CRM for estimating the Customer Lifetime Value of each football fan, using the powerful but simple Recency, Frequency and Monetary (RFM) model. To propose a potentially more suitable model for AFC Ajax, the weighted RFM alternative has been implemented, where the weights (relative importance) are determined using the Analytic Hierarchy Process (AHP) method, through the perception of domain experts within the football club. Then the K-means algorithm, one of the most widely used approaches in clustering methodology, is implemented to cluster the fans into segments based on similar lifetime values and loyalty. Each segment has further been ranked by using the simple weighted sum, resulting in multiple segments with a ranking based on the customer value and its priority. Finally, to evaluate and validate the proposed method, four viewpoints have been discussed. The first viewpoint is to compare the proposed weighted RFM model with the non-weighted RFM as baseline. Secondly, statistical models have been implemented to evaluate if the RFM variables significantly discriminate between the developed clusters. Thirdly, a predictive model is developed using the decision tree algorithm to assist in evaluating and validating the proposed model. And lastly, the same methodology has been applied to a non-football empirical case (online retail store based in the UK) for model appropriation. Furthermore, in order to maximize the performance of the potential outcome with regard to the time constraints and resource limitations, the scope of this study is limited to the usage of online business-to-consumer merchandising transaction data of each Ajax fan retrieved from the CRM. In the interest of achieving the objective of filling in both the research gap and the business gap within Ajax, the paper is structured around the following research questions:

• **Main research question:** To what extent can the weighted RFM model be used to cluster Ajax football fans based on their online merchandising purchasing behaviour?
• **Sub-question 1:** What is the performance quality of the proposed model when compared to the baseline?
• **Sub-question 2:** What are the main similarities and differences between the developed clusters?
• **Sub-question 3:** How does the proposed methodology perform when applied to an empirical case outside the football industry?

## 2 Related work

This section summarizes the most important existing literature related to this research.

### 2.1 Customer lifetime value (CLV)

The acquisition of new customers is crucial for the success of a company, but the retention of current customers is relatively more important, as a churned customer (*customer* that stopped using a company's product and/or service) equals to losing the whole profit that might be generated during a customer's lifetime (9). Along with incorporating new customers (10), has shown that loyal customers are more profitable than new customers. This conclusion is based on several behavioral patterns attributable to loyal customers: (1) loyal customers generate more profits because they get accustomed to the service and use the service more; (2) they are less price sensitive and thus, companies can charge more. Besides, a small increase in customer retention can lead to a significant boost in profits as well as translate improved sporting performances into enhanced business performances. The importance of commercialization in football from technology and data takes fandom to new heights and bring about new revenue generating opportunities for new potential customers (11). While grounded on these research, they collectively underscore that while acquiring new customers is crucial for business growth, retaining existing ones is relatively more important due to its profound impact on profitability, cost efficiency, and overall customer value.

Babak and Khanlari (2) concluded that it is crucial to first determine the profitability of a customer, as not all are equally financially interesting to the company. An approach where resources are implemented in line with the customer value may be a more suitable approach. This motivates companies to more effectively and efficiently fulfill the specific needs of their customers, by enhancing their marketing activities and eventually improving their profitability. A commonly used method based on a customer's profitability and loyalty is the Customer Lifetime Value (CLV). The CLV has been used by companies to improve their resource allocation and increase the performance of their marketing activities (12). Different models have been developed for estimating CLV. As proposed by Gupta et al. (13), examples include persistence models based on modeling behavior, probability models based on Pareto/NBD and Markov chains, diffusion/growth models based on customer equity, computer science models based on theory (i.e., utility theory) and econometric models. It however has been suggested by several authors (14–16) that the Recency, Frequency and Monetary (RFM) model primarily focuses on the most profitable customers, while the less profitable ones are avoided. This results in the resources being targeted at the customers which are financially most attractive to the company. While certain customers play a pivotal role in a company's market positioning and competitive strategy. These customers might include industry leaders, influencers, or early adopters whose endorsement can significantly boost the company's reputation. For instance, having a leading football player figure or a popular team as a customer can enhance brand credibility and attract new business, far outweighing the immediate profits from less strategically valuable customers. For instance, the relative importance of several predictors of nonprofit sports clubs’ success in recruiting new and keeping existing customers has been studied in Koenigstorfer & Wemmer (17) via a survey with managers from 284 German sports clubs, where they used random forest to determine the relative importance of predictors of member recruitment and retention in sports clubs, and to assess the nature of the relationships as they considers three innovation-related factors that have been shown to be relevant to the success of sports clubs in recruiting and retaining members. Wemmer et al. (18, 19) showed the positive effect of innovation activity on performance is driven by sports’ clubs use of the concept of coopetition. and use of outside knowledge, of which coopetition describes situations, in which organizations simultaneously cooperate and compete with the same organization (20). Nevertheless, the study of Buser et al. (21) applied an innovative three level model to a sample of 1,395 Swiss football club members selected from 138 teams across 42 clubs. They highlighted the importance of the team context, where pronounced goals of sporting success are detrimental, and a culture of mutual respect benefits member commitment. Accordingly, the team context should be included in theoretical and empirical models of member action in sport clubs.

Recently, a study on the impact of membership duration on the relationship between consumer knowledge and behavioral loyalty, specifically on assessing the mediating role of psychological engagement and consumers’ perceived service quality in the relationship between subjective customer knowledge (SCK) and behavioral loyalty among members of nonprofit sports service organizations (22). They found that direct effect indicates a significant influence of subjective knowledge on perceived service quality as perceived service quality significantly and positively influences psychological engagement and psychological engagement to be an important predictor of consumer behavioral loyalty. In addition, Akoglu and Ozbekthe (23) emphasized the importance of quality and trust in building customer loyalty for companies in the sports industry, which has been revealed that there is an important intermediary role of perceived quality and brand trust that manages the relationship between customers’ brand experience and brand loyalty as to Brand experience has a positive direct effect on perceived quality, brand trust and brand loyalty. On the contrary, Akoglu and Ozbekthe (24) encountered that a measure the impact of eWOM (electronic word of mouth) and brand celebrity use on the purchasing behavior of sports consumers was ineffective in their study.

In fact, the RFM model is one of the most powerful techniques to database marketing, as the model does not require any additional data of its customers while it is also relatively simple to develop in contrast to its alternatives (25–28). The following subsection further describes the RFM model and its application for customer segmentation using the CLV.

### 2.2 Recency, frequency & monetary (RFM)

The RFM model is based on the behavior of a customer and is commonly used to determine the CLV, segment the market, and evaluate the connection between company and customer. The RFM components are defined by Bult & Wansbeek (29) as follows:

• **Recency (R):** The period (i.e., in days) since the last purchase; a lower value corresponds to a higher probability of the customer making another purchase.
• **Frequency (F):** The number of purchases made within a time period; a higher frequency indicates greater loyalty.
• **Monetary (M):** The total amount of money spent during a period; a higher value indicates that the company should focus more on that particular customer.

The RFM is a generally applied model and has been deployed in various industries. It has been implemented in several applications including marketing, banking, retail, hospitality, and healthcare, but also non-profit organizations, public agencies, telecommunication, and transportation. Some of its applications are summarized in the following paragraphs. The work of Chan (3) used RFM to combine the targeting and segmentation of customers for marketing campaigns, resulting in a relatively better performance when targeting valuable customers using the proposed approach, in comparison with the random selection approach. The study of Babak and Khanlari (2) applied the RFM variables in combination with the K-means clustering algorithm, to develop a CLV model for clustering customers into different CLV segments. Cheng and Chen (4) describe an approach where the RFM features are combined with K-means, to develop classification rules and improve clustering accuracy. Kaymak (6) elaborates on how fuzzy clustering can be used to derive target selection models by using the RFM variables as features for distinguishing the customers. Lumsden et al. (8) implemented RFM in an all-inclusive travel vacation club, by using the model for clustering customers based on CLV using pre-purchase data of motivations for membership initiation. Li et al. (7) introduced a method for ISP companies in the telecommunication industry of Taiwan, where self-organizing maps were deployed to cluster customers into different segments with divergent usage activity patterns. The RFM model was then used to calculate the CLV of each segment and generate insights for the management to enhance their marketing strategies. Hsieh (5) proposed an approach where repayment behavior and RFM features were used, for a behavioral scoring model in combination with an integrated data mining model, to predict the groups with a high CLV of current credit card customers in a bank. The review analysis of Wei et al. (30) discusses several potential reasons why the RFM model is commonly used for customer segmentation in direct marketing. The first reason is that the RFM model is cost-effective in acquiring and quantifying essential customer behaviour analysis Kahan (14), and Miglautsch (15), where the data of customers and its transactions can be stored in an online available database. Secondly, the model is powerful in summarizing the purchase behaviour, as a relatively small number of variables are used, with a significant impact. This also may result in the application of RFM to be easily understood by decision makers and other stakeholders. Thirdly, the model can improve the profitability of a company in the short term, as the model is capable of effectively predicting the customers’ response (company's reaction to the queries and activities of the customer) Baecke & Van den Poel (31). Several disadvantages of RFM include that it primarily focuses on the current customers of a company, and technically cannot be applied to potential new customers, as the company does not have a purchase history of prospects (32). Another disadvantage is that the RFM provides limited insight into the scoring when customers do not buy frequently, spend often or have not purchased recently. This disadvantage may become problematic for companies where 80% of the revenue is generated by 20% of the customers (80/20 rule) Wang (33). This places new companies, who have customers that only purchased once with a small amount, in a disadvantaged position. To mitigate the disadvantages of the RFM model, researchers made attempts in developing other (improved) variants such as the RFMTC model by Yeh et al. (34), where the RFM model has been augmented using the elements Time (T) and Churn (C) probability using the Bernoulli sequence in probability theory, calling it the RFMTC model. Nonetheless, as customer-centric management does not necessarily imply that managers may not consider important data from products that can provide valuable insights, Heldt Rodrigo et al. (35) proposed an RFM/P model enables managers to have a more complete overview of future firm profits. Indeed, they focused on changes in customer purchase behavior regarding recency per product and frequency per product, therefore, RFM/P prediction accuracy was found to be better than traditional RFM.

Other variant examples include the RFD (Duration) model, RFE (Engagement) model and RFM-I (Interactions) model. A commonly used RFM variant is the weighted RFM model (WRFM), which is described in the next subsection. An example of the developed fan segments, where observations represent individual football fans.

### 2.3 Weighted RFM model (WRFM)

Instead of applying an equal weight for each RFM variable, Liu and Shih (36) introduced an approach where the Analytic Hierarchy Process (AHP) method was used to determine the relative importance of the weights for each RFM variable, resulting in an adapted weight for each variable. Since the weight is an influencing factor to the model, this variant is called the weighted RFM (WRFM). In contrast to the regular (and non-weighted) RFM model, one of the advantages of using the WRFM method in combination with AHP, is that it offers the possibility of adjusting the weights of the RFM model for each specific case in a particular industry. It offers a potentially enhanced model without the application of relatively complex methods, but rather the usage of a simple method in return for a minimal additional cost in terms of technical and human related resources. As the regular RFM model includes an equal weight for each RFM variable, the WRFM alternative can estimate a weight for each RFM based on the input of domain experts in the industry. This may result in a more powerful model for customer segmentation (37, 38). The AHP method is further described in the next subsection.

### 2.4 Analytic hierarchy process method (AHP)

The Analytic Hierarchy Process (AHP) method has been developed by Saaty (39) and is defined as a structured technique for organizing and analyzing complex decisions. Its application is commonly used for multicriteria decision-making. It is a robust method for using human input to solve integrated and fuzzy shaped problems. The AHP evaluates paired comparison judgments, using a fundamental scale of absolute numbers to give decision-makers the option to prioritize alternatives for a problem using an architectural structure. For each comparison, the decisionmaker assigns a number from 1 to 9. The method then measures the degree of consistency between the judgments. The judgments must be revised if the inconsistency degree of the judgments exceeds 0.1. For RFM analysis, AHP is commonly used to determine the relative importance (weights) of the RFM variables. The outcome of the approach results is a weight for each RFM, which then can be used to establish the WRFM model. To effectively apply the weighted RFM for customer segmentation, clustering algorithms may be used to implement this task. These algorithms are described further in the next subsection.

### 2.5 Clustering algorithms

Clustering is one of the data mining tools used to discover knowledge processes (40). Clustering algorithms aim to minimize the variance within groups, while maximizing the variance among groups. Many algorithms have been developed in clustering, such as K-means, fuzzy C-mean, hierarchical, and repetitive median K-means. Each clustering algorithm has its own advantages and disadvantages for each application. The K-means (41) has commonly and effectively been used for customer segmentation with the application of RFM analysis (2, 4, 16, 42). When K-means is applied for customer segmentation using RFM analysis, each RFM variable is first normalized using min-max normalization. The motivation behind this is that skewed input values may become problematic. After applying K-means for RFM analysis, the outcome is that each data point (customer) has been assigned to a particular cluster, which then can be used for evaluation purposes. A common way of evaluating and validating the results of RFM clustering analysis is by developing and training a decision tree classifier. The next subsection further elaborates on this approach.

### 2.6 Decision tree learning

Several studies have used the decision tree algorithm for the purpose of customer database segmentation (32, 42, 43). It is an important technique extensively used in classification. Advantages of the decision tree theory when used in RFM analysis include that it can produce understandable rules which can be used for the evaluation and validation of the (W)RFM model. It also can perform tasks without extensive computing, handle continuous variables in combination with categorical variables, and it can also learn which attributes are important for classification (44).

In RFM analysis, the decision tree algorithm also has been used for validating clustering results, where authors adopted the algorithm to construct a decision tree and rules to calculate the classification accuracy rates of existing customers. In RFM applications, the predictive model can furthermore be used for predicting the segments of potential customers and to which CLV ranking they may map to. A decision tree classifier that has been trained using the RFM model, can provide meaningful insights about how the clusters have been segmented and how well the clustering results can be used to predict each segment. Differences in class performance then can be evaluated to derive the strengths and weaknesses of the proposed model and its boundaries.

## 3 Methodology

### 3.1 Overview & motivation

There are many studies available regarding the use of clustering analysis for customer segmentation in various industries. There, however, is a lack of research in the football industry, resulting in a potential research gap. This study aims to positively contribute to this research gap, by avoiding significant deviations from the commonly used methods and techniques applied in the field. The aim of the methodology is therefore to adopt a setup which is in line with the state-of-the-art summarized in the previous section. This to make it possible for the findings of this study to be compared with other similar studies but originating from different industries. The proposed method of this study is summarized in (Supplementary Figure S1) and includes the use of the weighted RFM, together with AHP for determining the weights. The K-means clustering algorithm has then been used to cluster fans into segments, where the simple weighted sum of normalized RFM values is used to rank each cluster and identify the profitable customers. Finally, a decision tree classifier has been trained, to evaluate the predictability of each segment and generate opportunities for future studies in this field to relate their results with this study. The main motivation behind this approach is that the combination of using WRFM together with AHP, K-means for clustering and decision trees for evaluation, is a proven and effective setup that has commonly been used in various industries to cluster customers into segments and evaluate and validate its findings. The setup also does not require many (costly) resources, while the return may have a large potential impact. The application of this setup is relatively simple with a powerful potential outcome. The stated approach furthermore contains all the required methods and techniques to successfully achieve the objectives of this study within its scope and time constraints. Additionally, the findings produced by the application of AHP, K-means and decision tree classifier on the WRFM model, results in findings that are relatively simple to explain and adopt, which may increase the potential impact of the findings on improving the marketing activities of Ajax as the facilitating company. Finally, as this proven setup is commonly used in other studies, it makes it possible to compare the findings of this research with earlier and future studies, intending to positively contribute to the research gap in the football industry.

### 3.2 Data preparation

The used transaction data for each Ajax fan is retrieved from the Ajax CRM and contains all the merchandising transactions originating from the online webshop (45). After collecting the data, exploratory data analysis (EDA) has been performed to inspect, evaluate and understand the dataset and its distributions. These insights then have been used to make decisions regarding the preparation of the dataset, which are summarized in the upcoming paragraphs. First of all, columns that have not been used for this study have been excluded. The resulting dataset includes the following six columns: *orderNumber, orderStatus, customerID, financialStatus, orderAmount* and *orderDate*. Each record represents a transaction (order) of an Ajax fan. The columns include specific details regarding the transaction. The dataset then has been filtered on transactions with a *paymentStatus* of “*paid*”, and an *orderStatus* of “*shipped*”, to avoid the inclusion of cancelled or unpaid transactions and only involve records that contribute to the CLV of a fan. Records that contain at least one empty value have also been removed, to avoid the usage of low-quality data. Internal transactions used for the registration of stock movements from one warehouse to the other, also have been removed by excluding *customerID's* that are related to Ajax departments. Moreover, unreasonable transactions such as orders with negative *orderAmounts* also have been excluded.

The dataset then has been filtered to a time range of exactly five football seasons, starting from the 1st of July 2015 up to and until the 30th of June 2020, as these are the starting and end date of a regular football season of Ajax. The motivation behind the time range filter is that the merchandising performance is strongly correlated with Ajax's football performance in matches and competitions. A season where Ajax participated in the Champions League, results in significantly higher revenues than a season where Ajax lost all competitions and did not participate in the Champions League. The specified range of season 2015/2016 up to and until season 2020/2021 includes a balanced football performance of Ajax, including both high and low-performance seasons. Limiting the dataset to these five seasons may result in a more representative dataset which can be used for customer segmentation in current and future seasons.

After evaluating the outlying data points through visual inspection and EDA (Supplementary Figures S2), by inspecting the fans with large transactions, it has been concluded that these “outliers” are genuine Ajax fans that intensively have been interacting with Ajax. As the non-transactional records such as internal stock movements have been left out, the resulting dataset only includes genuine transactions that are representative for current and future Ajax fans. For this reason, the decision has been made to not exclude the “outlying” transacting fans, in order to develop a realistic model that more accurately reflects the fan base of Ajax. This may come at the cost of decreased performance of the model. The representivity of the model considering the Ajax fans and its analyzability however has a higher priority.

### 3.3 WRFM model development

#### 3.3.1 RFM feature extraction

In order to develop the RFM model, first the RFM features have been extracted from the dataset. This has been done for each unique Ajax fan in the dataset, where the Recency has been calculated by counting the number of days between the fan's last order and the last day of the dataset (30-06-2020). The Frequency has been calculated by taking the total order count. The Monetary has been calculated by taking the total sum of *orderAmount*. This results in an aggregated dataset, where each record represents an Ajax fan, and the columns the developed RFM features.

#### 3.3.2 Feature normalization

Next, to maximize the performance of the K-means clustering, the RFM values have been normalized. This has been implemented by using the profit form (Equation 1) and cost form (Equation 2). Notably, *x*’ and *x* represent the normalized and original R, F and M values, respectively, while *x^L^* and *x^S^* represent the largest and smallest R, F and M values of all fans. Since the variables Frequency and Monetary positively influence the CLV or loyalty of a fan, as a higher value represents a higher CLV, these variables have been normalized using the following profit form:

(1) $$x^{\prime }\;=\;(x\;-\;x^{S})/(x^{L}-x^{S})$$

In contrast, the Recency variable negatively impacts the CLV, as a higher value represents a larger gap since the last transaction. For this reason, the Recency variable has been normalized using the cost form, where a higher normalized value reflects a smaller gap since the last transaction.

(2) $$x^{\prime }\;=\;(x^{L}\;-\;x)/(x^{L}-x^{S})$$

#### 3.3.3 Determining relative importance of weights

The AHP method then has been applied for determining the relative importance of the weights for each RFM variable. This has been implemented by interviewing eight domain experts within Ajax to judge the RFM weights. The following candidates from Ajax participated in the AHP: Chief Data Officer, Digital Transformation Officer, Manager Webshop, Database Marketing Manager, Digital Marketing Specialist, Marketing Analyst, CRM Marketeer and Data Scientist.

Each evaluator has been invited for a 1-on-1 interview with the author, where the evaluator was asked to assign a score between 1 and 9 based on AHP's fundamental scale of absolute numbers (Supplementary Table S1), for each of the following pairwise combination: Recency vs. Frequency, Recency vs. Monetary and Frequency vs. Monetary. All answers have been collected to assess the consistency of the pairwise judgments, where the answers have been revised where necessary to achieve a consistency degree of no more than 0.1. Finally, the Eigenvalue computation has been used to derive the relative weights of each RFM variable. The outcome resulted in a weight for each RFM variable, *w_R_*, *w_F_* and *w_M_*, respectively, based on the aggregated assessment of the evaluators. Each normalized RFM variable then has been multiplied by its respective weight, in order to derive the weighted normalized RFM values.

#### 3.3.4 K-means clustering

The weighted normalized RFM variables then have been given as input to the K-means algorithm. To implement K-means, the number of clusters, *k*, need to be specified in advance. In this study, the parameter *k* has been set to 8. The motivation behind the eight clusters, is that for each cluster it then can be evaluated if its non-normalized R, F and M value is above or below the total average of that particular variable, where the symbols ↑ (above average) and ↓ (below average) then can be assigned for each cluster. Next, a pattern can be established by concatenating the specified symbol of each variable. Example: a cluster with a below average Recency, and above average Frequency and Monetary, results in the following pattern: R↓ F↑ M↑. Enhancing this approach results in eight (2 × 2 × 2) possible combinations.

The advantage of this approach is that meaningful insights can be linked to each segment by identifying and evaluating the pattern of each cluster. An alternative to this approach is to use heuristics, such as the Partition Coefficient Index (PCI), Dunn Index (DI) or the “Elbow” method, to determine the optimal number for parameter *k*. When applied to the currently used setup, the alternative approaches however only resulted in three to four clusters, which significantly limits the analyzability and quality of the actionable insights that may be derived when compared to using eight clusters. Additionally, the used dataset includes a large amount of Ajax fans, where clustering the fans in only three to four clusters may significantly restrict the improvability of the marketing activities that may be used to target and approach the newly created segments. Using eight clusters provides sufficient granularity to more accurately target and align the marketing strategy with the specific needs of each cluster. This decision again may come at the cost of a decreased model performance. However, the potentially improved quality of actionable insights due to the higher number of clusters comes at a higher priority.

#### 3.3.5 CLV ranking of clusters

The outcome of the K-means is a set of eight clusters, where each Ajax fan has been assigned to one cluster. The CLV of each cluster then has been calculated using Equation 3. Notably, each cluster is denoted as $C_{R}^{j}$, $C_{F}^{j}$ and $C_{M}^{j}$, respectively for *j* = 1 to *k* (number of clusters). $C_{R}^{j}$, $C_{F}^{j}$ and $C_{M}^{j}$, are computed by averaging the normalized RFM values of the fans in cluster *j*. Let $CLV^{j}$ be the CLV of cluster *j*. $CLV^{j}$ is computed as the weighted sum of $C_{R}^{j}$, $C_{F}^{j}$ and $C_{M}^{j}$, where *w_R_*, *w_F_* and *w_M_* are the weights of the RFM variables determined by AHP.


(3) $$CLV^{j}=C_{R}^{j}\;w_{R}+C_{F}^{j}\;w_{F}+C_{M}^{j}\;w_{M}$$

The CLV ranking is then derived according to the cluster's calculated CLV. The ranking is assigned in ascending order, where the cluster with the highest CLV gets assigned the rank of 1 (top priority) and the lowest the rank of 8 (least priority). Finally, based on the ranking and pattern, one of the following names is assigned*: Golden Fan, Promising, Needs Attention, Churned/Low Value and New Fan*. The segment names are inspired by both the football industry, and the commonly used names in the field of RFM analysis.

### 3.4 Evaluation & validation framework

The evaluation and validation of the results are structured around the research questions. The following paragraphs describe the evaluation and validation methods divided per sub-question.

#### 3.4.1 Performance of proposed model vs. baseline (sub-question 1)

In order to evaluate and validate the performance of the proposed model, a baseline has been developed. The baseline has been implemented by taking the non-weighted RFM model (NW-RFM), where the weight of each RFM variable is assumed equal. The CLV ranking then has been assigned using the non-weighted sum of the RFM variables, resulting in a CLV rank for each cluster of the baseline model. The comparison is then performed by comparing if and which cluster(s) differ between the proposed and baseline model and elaborate on the findings. The CLV rank and pattern of the clusters are also taken into consideration during the comparison, to specify any differences more accurately between the models. To further validate the proposed method, a decision tree classifier has been trained to evaluate if and how well the RFM variables can be used to predict each cluster. For the development of the classifier, a randomized split has been made to assign 70% of the data as the training set and the remaining 30% for the test set. A maximum tree depth of 5 nodes has been enforced to avoid overfitting and keep the tree relatively simple for explainability purposes. The Gini Index has been used as a split criterion over the Information Entropy alternative, as it showed relatively better performance.

The evaluation metrics used to evaluate the classifier's performance for each cluster have been shown below in Equations 4–7, where *TP*, *TN*, *FP*; and *FN* represent the True Positives, True Negatives, False Positives and False Negatives, respectively. To evaluate the performance of the overall model, the overall accuracy has been used, along with the macro and weighted average scores of the precision, recall and F1-score.

(4) $$Precision\;=\;\frac{TP}{TP+FP}$$

(5) $$Recall\;=\;\frac{TP}{TP+FN}$$

(6) $$Accuracy\;=\;\frac{TP+TN}{TP+TN+FP+FN}$$

(7) $$F_{1}=2*\frac{Precision*Recall}{Precision+Recall}$$

The motivation behind the mentioned evaluation metrics is that these metrics are commonly used to evaluate classifiers and provide reasonable insights in the performance of the class predictions. By evaluating the performance of each cluster and the overall model performance, insights can be derived regarding the performance quality and accuracy of the proposed model.

#### 3.4.2 Similarities and differences between clusters (sub-question 2)

With regard to sub-question 2, first a statistical Analysis of Variance (ANOVA) has been executed to evaluate if the RFM variables significantly discriminate between the clusters of the proposed model. Then a *post-hoc* test has been performed to evaluate which exact clusters are (not) significantly discriminated using RFM. Finally, to derive the similarities and differences between the clusters, the non-normalized and non-weighted RFM values of each cluster have been analyzed. These are reflected in light of the established CLV ranks and patterns, to derive what the main contributors may be for clusters to be related or distinct from one another.

#### 3.4.3 Method performance on empirical case (sub-question 3)

With the objective to compare and validate the performance of the proposed methodology on an empirical case outside the football industry, first the exact same methodology has been applied to a dataset from a UK-based and registered online retail store, mainly selling unique all-occasion gift-ware. Most of its customers include wholesalers. The dataset includes real-life online retail transaction data and has been released by the London South Bank University Chen (46) for educational purposes. The motivation for selecting this particular dataset is due to the fact that both datasets involve an online webshop, while the empirical data set originates from another environment with different transactional dynamics. It also involves different distributions of the data. Evaluating the effect of the proposed methodology on a dataset which strongly differs from the Ajax dataset is an interesting comparison to analyze, this is to further assess to which extent the methodology performs on a non-football related dataset.

Furthermore, as it is not possible to perform AHP for the empirical case due to time and resource constraints, the RFM weights are determined by the assumptions of the author, based on analyzing the dataset and taking the motive of the online store and its customers into consideration. After implementing the same methodology on the empirical data set, its results are compared with the results of the proposed model. This is done in two ways: first the patterns of the empirical case's developed clusters are compared with the patterns of the proposed model's clusters, to evaluate to which extent the assigned CLV ranks and segments differ from one another. Secondly, the classifier performance of each model is compared, to reflect if the results (strongly) differ from one another.

## 4 Results

This section summarizes the methodology's most important results. The evaluation, along with the conclusion for each sub-question, is done in Section 5 Evaluation & Conclusion.

### 4.1 Ajax merchandising transaction data

After implementing the preparation steps of subsection 3.2 Data Preparation on the original Ajax dataset, it resulted in 500,591 transactions and 6 columns. The dataset, where the RFM variables have been extracted by aggregating the transactions for each fan, consists of 315,916 fans and 4 columns. An anonymized and non-normalized sample of the dataset is shown in (Table 1). In the summary statistics (Supplementary Table S3) and a visualization of the dataset distributions (Supplementary Figure S5) can be found. A general finding observed from (Supplementary Table S3) includes that on average, fans do have a reasonably high spending per purchase, but do not purchase often and have not purchased recently. Another observation using (Supplementary Figure S5) is that each RFM variable is in relation to the other, where a higher value of one variable corresponds to a higher collective RFM value.

**Table 1:** Anonymized and non-normalized sample of the dataset, showing aggregated RFM values for each Ajax fan.

Customer ID *(anonymized)*	Recency	Frequency	Monetary
*********	946	1	33.43
*********	90	4	321.52
*********	1,701	1	18.87
*********	233	1	21.03
...	...	...	...
*********	317	2	176.28

Source: author's own data analysis using dataset (49), refer to Methodology section for further details.

### 4.2 AHP relative feature importance

The anonymized findings of the Analytic Hierarchy Process (AHP) are summarized in (Supplementary Table S2). What can be concluded from the calculated relative RFM weights of 0.409 for Monetary, 0.343 for Frequency and 0.248 for Recency, is that the eight participants put the highest importance on Monetary, then on Frequency and the least on Recency. The degree of consistency between the judgments does not exceed more than 0.1, which means that the judgments are acceptably consistent. During the interviews, a few explanations by the participants have been observed by the author that may substantiate the AHP results. Firstly, fans that spend more and purchase less often, are more preferred than fans who buy often but for smaller amounts. This generates more revenue from products with a relatively higher price and profit margin, than cheaper products with a relatively lower profit margin. Secondly, fans that purchase often are preferred over those who have recently purchased, because most fans often spend only once per year, for example during the release of the new Ajax shirt of the new season. On the contrary, some participants mentioned that the fans who have recently purchased are more important than those who frequently buy and have large spending, because in their opinion a healthy relationship between Ajax and its fans is not only expressed monetarily, but also how strongly the fan interacts with Ajax in terms of (digital) content, communication, and event participation. A general observation is that participants with a management related position seem to put a higher importance on the Monetary aspect of a fan, while those with an employee-related position seem to more prioritize the Recency or Frequency element of a fan. This (partially) may be explained due to the fact that from one side the multi-annual strategic plan of Ajax emphasizes a significant increase in operational profitability, resulting in a stronger focus on generating more revenues. While on the other hand, the non-managers are relatively more inclined to reason from the perspective of the fan, as they are relatively closer to the fan due to their daily work.

All in all, the shared opinions between participants are therefore divided. The overall group result represents the shared opinion of all participants, which then is quantified and used as input for the development of the weighted RFM model.

### 4.3 Clustering analysis of football fans

The clustering results of both the proposed (weighted RFM) and baseline (non-weighted RFM) models are visualized in (Table 2), which then is used for comparing the performance of the proposed model with the baseline. For each cluster, finally an RFM pattern has been derived by evaluating if the average RFM value is above or below the total average of the variable. These results are summarized in (Table 3) along with the non-weighted and non-normalized RFM values of each cluster.

**Table 2:** Clustering result of the proposed and baseline model.

Cluster no.	# Fans	Proposed model weighted RFM				Baseline model non-weighted RFM			
		R	F	M	WRFM & rank	R	F	M	NW-RFM & rank
3	10,576	0.203	0.286	0.224	0.713 (1)	0.817	0.835	0.548	2.22 (1)
4	15,410	0.196	0.128	0.22	0.544 (2)	0.791	0.373	0.537	1.701 (2)
1	31,817	0.21	0.103	0.083	0.396 (3)	0.845	0.301	0.203	1.349 (3)
6	15,593	0.096	0.094	0.112	0.302 (4)	0.386	0.274	0.273	0.933 (5)
5	47,124	0.2	0.000	0.092	0.292 (5)	0.805	0.000	0.226	1.031 (4)
2	95,275	0.203	0.000	0.025	0.228 (6)	0.820	0.000	0.061	0.881 (6)
0	64,937	0.113	0.001	0.037	0.151 (7)	0.457	0.003	0.09	0.550 (7)
7	35,184	0.033	0.001	0.053	0.087 (8)	0.134	0.004	0.13	0.268 (8)
Total average	39,490	0.157	0.077	0.106	–	0.632	0.224	0.258	–

For each cluster the total fan count is shown, along with the mean of the normalized RFM values, where the RFM of the proposed model are adjusted using the AHP weights. The simple weighted sum of the RFM, along with the CLV ranks, are shown in ascending order. The differences between the models are marked with a darker color. Source: author's own data analysis using dataset (49), refer to Methodology section for further details.

**Table 3:** Overview of the proposed model's clustering results.

Cluster no.	Segment	# Fans	R (days)	F	M (Euro)	Pattern	WRFM rank	NW-RFM rank
3	Golden fan	10,576	334.99	4.34	229.52	R↓ F↑ M↑	1	1
4	Golden fan	15,410	383.12	2.49	224.78	R↓ F↑ M↑	2	2
1	Promising	31,817	284.34	2.2	85.8	R↓ F↑ M↓	3	3
6	Needs attention	15,593	1,122.55	2.09	114.99	R↑ F↑ M↑	4*	5*
5	New fan	47,124	357.64	1	95.27	R↓ F↓ M↓	5*	4*
2	New fan	95,275	329.3	1	26.46	R↓ F↓ M↓	6	6
0	Churned/low value	64,937	992.81	1.01	38.57	R↑ F↓ M↓	7	7
7	Churned/low value	35,184	1,582.45	1.02	55.11	R↑ F↓ M↓	8	8
Total average	–	39,490	673.40	1.89	108.81	–	–	–

For each cluster the average non-weighted and non-normalized RFM values are shown. For each RFM value the pattern has been determined by evaluating if it is above or below the total average. Based on the pattern a segment name then has been assigned. The ranks of the weighted and non-weighted RFM are also shown. The light cells with a star (*) mark the differences between the models. Source: Author's own data analysis using dataset (49), refer to Methodology section for further details.

### 4.4 Decision tree performance

The performance of the decision tree classifier is summarized in (Table 4). The confusion matrix is shown in (Supplementary Table S9).

**Table 4:** Performance results of the decision tree classifier.

Decision tree classifier results (*N* = 94,775)					
WRFM rank	Accuracy/error	Accuracy rate (%)	Precision	Recall	F1-score
1	3,014/34	98.88%	0.99	0.95	0.97
2	4,341/280	93.94%	0.94	0.93	0.93
3	9,169/272	97.12%	0.97	0.96	0.96
4	4,058/588	87.34%	0.87	0.87	0.87
5	13,925/285	97.99%	0.98	0.99	0.98
6	28,501/150	99.48%	0.99	0.99	0.99
7	19,033/1,136	94.37%	0.94	0.98	0.96
8	9,841/148	98.52%	0.99	0.94	0.96
Total:	91,882/2,893	Macro average:	0.96	0.95	0.95
Accuracy (%):	96.95%	Weighted average:	0.97	0.97	0.97

For each cluster, the (in)correctly predicted classes are shown, along with the accuracy rate (%), precision, recall and F1-score. The overall performance is specified in the bottom as overall (in)correctly predicted classes, accuracy rate (%) and macro/weighted averages. Source: Author's own data analysis using dataset (49), refer to Methodology section for further details.

### 4.5 Statistical tests for analysis of variance

In order to test whether the RFM variables significantly discriminate between the clusters, first a Shapiro-Wilk Test of Normality was executed for each RFM to assess if the variables come from a normal distribution. The results are shown in (Supplementary Table S4) and Q-Q plots can be found in (Supplementary Figure S6).

With an *a* (alpha) of 0.05 and a *p*-value of <0.05, the null hypothesis that the RFM variables are normally distributed is rejected. This means that we may not assume normality for the distributions of the RFM variables. As the Analysis of Variance (ANOVA) is a parametric test which assumes normality, the nonparametric alternative Kruskal-Wallis *H* test has been chosen. The null-hypothesis *H*_0_ and alternative hypothesis *H*_1_ are as follows:

• *H*_0_: Population medians are equal.
• *H*_1_: Population medians are not all equal.

The results of the Kruskal-Wallis H test for each RFM is summarized in (Supplementary Table S5). With a *p*-value for each test being lower than the *a* of 0.05, the null-hypothesis that the population medians are equal, is rejected for each RFM variable. This means that we may assume that the RFM variables may be used to distinguish between the eight clusters. After the Kruskal-Wallis H test, the *Post-Hoc* Dunn's Test was implemented to determine which exact cluster significantly differs from one another using the RFM. The findings are shown in (Supplementary Table S6–8). Using an *a* of 0.05, it can be concluded that the null-hypothesis for the tests that there are no differences between the clusters can be rejected, except for the following cluster combinations: clusters with WRFM ranks 2 vs. 5 for Recency, and 5 vs. 6 and 7 vs. 8 for Frequency. This means that the RFM variables may significantly discriminate between the clusters, and possibly not for the insignificant cluster combinations. The latter may (partially) be explained by the fact that the Recency in cluster ranks 2 and 5, only slightly differ from each other: with 383.12 and 357.64 for clusters 2 and 5. The same may apply for the Frequency variable: 1.00 and 1.00 for clusters 5 and 6, and 1.01 and 1.02 for clusters 7 and 8. An important note for all performed tests is that the statistical significance in the sample is not due merely to random sampling variation, but reflects an actual difference or relationship in the population. Practical importance is a substantial difference or relationship which is important to determine, as the *p*-value significance does not determine the practical importance of the result, but it may help to support the decisions being made.

### 4.6 Empirical case for model appropriation

The findings of applying the proposed methodology on the online retail store dataset (46) is summarized, where the summary statistics of the dataset are shown in (Supplementary Table S10), a graphical visualization of the dataset in (Supplementary Figure S7), the clustering results in (Supplementary Table S11) and the classification performance in (Supplementary Table S12). An observation regarding the empirical dataset in contrast to the Ajax dataset, is that customers buy more often and for larger amounts. The mean Recency is relatively lower, where the Frequency and Monetary are relatively higher. As the dataset originates from an online retail store mainly selling all-occasion gift-ware, where most of the customers include wholesalers, the expected average price of a product is relatively low. Based on the assumptions of the author, the weights for the RFM variables therefore are 0.8 for Frequency and 0.1 for both Recency and Monetary. The motivation for those weights is that wholesalers mainly buy in bulk to generate economies of scale, so they can further resell the products for a higher sale price. The highest importance therefore falls on the Frequency. Recency and Monetary have been given an equal and relatively low importance, as a retailer who primarily sells to wholesalers, might be mainly interested in selling in large volumes to further minimize its operational cost and generate larger profits by selling in higher quantities. Customers who spend relatively more but less often, or those who have purchased recently compared to those who have purchased some time ago, are significantly less important than those who purchase continuously. The main difference when comparing the Ajax clusters to the clusters of the empirical dataset, is that three out of eight clusters differ from one another. Another finding is that the two segments *Promising* and *Needs Attention* from the Ajax dataset, do not exist in the list of clusters of the empirical dataset. Furthermore, the *Golden Customers* in the empirical dataset spend significantly more than the overall average, where the *Churned/Low Value* spend significantly less and less often.

## 5 Evaluation & conclusion

The following subsections will now further evaluate the findings and draw conclusions structured by each sub-question.

### 5.1 Overall clustering analysis

In (Table 3) each cluster represents a market segment. Fans in WRFM cluster ranks 1 and 2 have the same characteristic with a pattern of R↓F↑M↑, where the clusters have an average Recency of 334.99 (cluster 1) and 383.12 (cluster 2), which is above the total average of 673.40 and a Frequency (4.34 and 2.49) and Monetary (229.52 and 224.78) exceeding the total average Frequency of 1.89 and Monetary of 108.81. Hence, clusters 1 and 2 would be considered the *Golden Fans*, as they are the loyal customers who purchase frequently and contribute the most merchandise monetary value to the club. *Golden Fans* are the biggest and most important contributors to the company's business-to-customer merchandising profitability. Efforts should be made to maintain the fan's activity, for example by providing special services or a loyalty program to stimulate, ensure and reward the fan's loyalty and behavior. Cluster 3 displays the pattern R↓F↑M↓ where the average Recency is the lowest of all segments, with an above average Frequency, but below average Monetary. These fans have a strong potential of becoming a *Golden Fan*, they however need to increase their spending. For this reason, this segment is called Promising. Marketing efforts should aim to identify the specific needs of this segment, for example by providing them with merchandise that better matches their desires. Another effort that could be made is making the fans realize that increased spending will result in becoming qualified for special services and/or a loyalty program. This should stimulate this segment to increase their monetary spending, in order to motivate them to upgrade to a *Golden Fan*. Cluster 4 arrays the pattern R↑F↑M↑, and may represent fans who once enjoyed a decent relationship with the club or even have been a Golden Fan. Their purchase Frequency and quantity exceed the total average, they however have not made any transactions lately. These fans may churn if the club “ignores” them. This segment therefore is called *Needs Attention*. An important task regarding this segment is to evaluate whether inattentiveness towards those fans triggers the “withdrawing” behavior, which for example may be caused by not providing relevant and sufficient products in the store for the type of football fan. An Ajax fan enjoying the season with his partner and children (family segment), may have other merchandise preferences than one fan who shares the experience with his friends (group of friends segment). Where fans from the family segment may purchase more child-related products, the fans from the group of friends segment most likely may not. Clear strategies need to be identified in order to retain those fans, as retaining an existing customer is significantly cheaper and financially more profitable than developing a new one (47). Clusters 5 and 6 show the pattern R↓F↓M↓ and may represent the *New Fans* who have recently visited Ajax to make a (first) purchase. Although their Monetary contribution, their purchase Frequency and Recency were all below the total average, which may indicate that these fans may try to establish a closer relationship with the club. Or, simply are the fans that once purchased gifts for their family and/or friends. These fans however have the potential to one day become a Golden Fan. Efforts therefore should be made to make these fans realize that if they increase their purchase Frequency, the club may provide them with improved services like the *Golden Fans*. The club also should try to attempt to develop a long-term relationship with the *New Fans*, by evaluating and fulfilling the specific needs of those fans. Finally, clusters 7 and 8 display the pattern R↑F↓M↓, where the average purchase has been relatively long ago, while they do not purchase frequently nor for relatively higher amounts. These fans have been given the name Churned/Low Value, as these fans either churned or simply contribute a relatively low merchandise value to the club. They probably minimally visit the webshop and make relatively low amounts of transactions. These fans also may only make transactions during sales, for example during the reduced price for last season's collection. The club may attract these fans by reducing prices, it however may suffer from reduced margins as a result. These fans therefore have the lowest relative importance when compared to the other segments. Hence, this analysis revealed distinct market segments among fans, notably the “Golden Fans” who are highly loyal and contribute significantly to the club's revenue. Efforts should focus on retaining their loyalty through tailored services. The “Promising” segment shows potential for growth but requires strategies to increase spending. “Needs Attention” fans, once loyal, need re-engagement strategies to prevent churn. “New Fans” represent opportunities for future growth with targeted efforts to increase engagement.

### 5.2 Weighted vs. non-weighted RFM

***Sub-question 1: What is the performance quality of the proposed model when compared to the baseline?*** When comparing the clustering result of the weighted RFM (proposed model) to the non-weighted RFM (baseline), it can be concluded that there is a difference in the ranking of the clusters *Needs Attention* and *New Fan*. Where the WRFM model ranks these clusters as rank 5 for *Needs Attention* and rank 6 for *New Fan*, the baseline assigned a different ranking, namely placing the *New Fan* at a higher priority. As can be concluded from the weights determined by AHP, the decision makers of Ajax place the highest importance on Monetary, then on Frequency and the least on Recency. As the segment *Needs Attention* has a higher Monetary and Frequency than *New Fan*, only the Recency is worse (as a higher Recency leads to a lower normalized Recency using the cost form, which then is used for determining the CLV rank). This means that it is reasonable to place the *Needs Attention* segment on a higher rank than *New Fan*, which only has a higher ranking in the baseline due to the strongly better Recency and an almost similar but lower Monetary. The difference may be backed by the fact that it is more suitable to retain a current customer than to allocate resources in acquiring and developing a new customer (47). Thus, placing the *Needs Attention* cluster on a higher priority than *New Fan* is therefore more appropriate considering Ajax's situation. It therefore may be concluded that the weighted RFM model may be a better approach in evaluating the CLV rank of a cluster, when compared to the non-weighted RFM alternative. Hence, this study compared the performance of weighted and non-weighted RFM models, demonstrating the superiority of the weighted approach in prioritizing segments accurately, aligning with the club's objectives. This highlights the importance of considering different weights for RFM variables in segment evaluation.

### 5.3 Evaluation of classification performance

From (Table 4) can be concluded that the classifier is performing reasonably well by using the RFM variables in predicting the clusters. From the total 94,775 observations in the test set, the classifier manages to classify 91,882 (96.95%) in the correct class, with an error of only 2,893 cases (3.05%) and relatively balanced macro and weighted average scores. An interesting observation is that nearly all clusters have relatively similar performance, except for the middle boundary cluster 4, which only has an accuracy of 87.34% and precision, recall and F1-score of 0.87. When evaluating the confusion matrix shown in (Supplementary Table S9), it can be concluded that out of the total 4,647 predictions, the classifier wrongly classified rank 4 for rank 3 (242 cases), rank 1 (165 cases), rank 2 (93 cases), rank 8 (75 cases) and rank 7 (13 cases). This may be caused by the fact that rank 4 exactly is in the middle of all the clusters with an average Frequency and Monetary close to the overall average, which may confuse the classifier. The slightly above average Frequency and Monetary values may confuse the classifier in predicting them for a higher class, while the high Recency may confuse the classifier in predicting the lowest segments (ranks 7 and 8). These findings may be confirmed by evaluating the other ranks in the confusion matrix, as they are also commonly incorrectly classified as rank 4. Hence, the classifier demonstrated strong performance in predicting segments, with a high accuracy rate. However, challenges were observed in accurately classifying the “middle boundary” cluster, indicating potential areas for improvement in classification strategies.

### 5.4 Cluster similarity analysis

***Sub-question 2: What are the main similarities and differences between the developed clusters?*** One of the main similarities that can be derived from the clustering results, is that the segments *Golden Fan*, *Promising* and *New Fan* all have in common that they have a below average Recency. This means that it may be worth it to allocate marketing resources to those fans, as they are currently unlikely to churn. The segments *Needs Attention* and *Churned/Low Value* however have an above average Recency, which both require selective and specific treatment, to reactivate them in becoming a transacting fan again.

Another similarity includes that all segments frequently purchase above average, except for the segments *New Fan* and *Churned/Low* *Value*. Assessing a lower priority for those two segments is therefore a reasonable approach. Furthermore, the *Promising* segment has a similar below average Monetary behavior, like the segments *New Fan* and *Churned/Low Value*. It, however, has a higher priority than the other two segments, as it has a very low Recency with an above average Frequency, meaning that it is not churned and more likely to become a Golden Fan, even though the current Monetary spending is below average. Allocating marketing resources to the *Promising* segment therefore may potentially result in a higher return on investment than when those are allocated to the segments *New Fan* or *Churned/Low Value*. Main differences include that the two *Golden Fan* segments differ from each other in terms of purchase Frequency, where cluster 1 has an average Frequency of 4.34 and cluster 2 of 2.49. This may mean that cluster 1 purchases more often, but for smaller amounts, whereas cluster 2 purchases less often, but for relatively larger amounts. Cluster 1 therefore is more loyal, as they purchase more often, whereas cluster 2 may result in a larger return on investment, as a fan spends relatively more on a single purchase. There also is an interesting difference in the two *New Fan* segments, where cluster 5 has an average Monetary spending of 95.27 and cluster 6 of 26.46. This difference may be caused by a potential distinction between the new fans that purchased an Ajax jersey once, while the other may have purchased one or more smaller products such as an Ajax scarf or cap before the match. It therefore is a reasonable approach to assign a higher priority to the new fans that have the highest spending. Another difference is between the two *Churned/Low Value fans*. Here the Frequency and Monetary are relatively equal, where only the Recency significantly differs from each other: 992.81 days for cluster 7 and 1,582.45 for cluster 8. It therefore is reasonable to place these two clusters at the lowest priority, where the cluster with the highest Recency of all clusters is placed at the bottom, as they churned a (very) long time ago.

Therefore, similarities and differences among clusters were identified, guiding targeted marketing efforts. Notably, understanding differences in purchase behavior among segments can inform resource allocation for maximum impact.

### 5.5 Applicability on empirical case

***Sub-question 3: How does the proposed methodology perform when applied to an empirical case outside the football industry?***

After applying the proposed methodology to the empirical case, it can be concluded that three out of eight clusters are different from the Ajax WRFM model. This may be explained by the fact that Ajax fans generally purchase less often and for smaller amounts when compared to the customers from the online retail store. Customers from the latter purchase larger quantities for higher amounts. The unit price, however, may be significantly lower, as the store mainly sells gift-ware primarily to wholesalers. Furthermore, when evaluating the classification performance of the decision tree classifier trained on the empirical dataset (Supplementary Table S12), it can be concluded that the classifier is reasonably accurate in predicting each class. With an accuracy of 99.09% and average macro/weighted precision, recall and F1-score of 0.99, it even can be concluded that the classifier performs relatively better than the classifier trained on the Ajax data. This partially may be explained by the fact that the RFM variables of the empirical dataset have wider distributions than the Ajax dataset, causing relatively more variability between the average RFM values of the clusters, making it easier to distinguish clusters from one another. This is in contrast to the Ajax dataset, where the differences in the average RFM values of the clusters are relatively smaller. All in all, the findings show that three out of eight of the developed clusters differ when the methodology is applied to a non-football empirical case with different dynamics in purchase behavior. Therefore, when applied to a non-football empirical case, the methodology yielded valuable insights, albeit with some variations in cluster composition. This suggests the methodology's potential applicability beyond the football industry, albeit with adaptations to suit different contexts.

### 5.6 Summarization of marketing recommendations for football clubs

Based on the findings of this research, below a brief summary of the primary marketing recommendations for football clubs.

1. Maintain Golden Fans’ Loyalty:
  • Provide special services or loyalty programs to reward frequent purchasers.
  • Offer exclusive merchandise and personalized experiences to sustain engagement.
2. Increase Spending in “Promising” Fans:
  • Identify and cater to specific needs and preferences.
  • Highlight benefits of increased spending, such as eligibility for special services or loyalty programs.
  • Offer targeted promotions and incentives to encourage higher spending.
3. Re-engage “Needs Attention” Fans:
  • Investigate reasons for reduced engagement and address them, such as by expanding product variety.
  • Tailor marketing strategies to different fan segments, like families or groups of friends.
  • Reconnect with personalized offers and experiences to prevent churn.
4. Develop Long-Term Relationships with “New” Fans:
  • Communicate the benefits of regular purchases, such as enhanced services.
  • Understand and meet the unique needs of new fans to foster loyalty.
  • Use initial interactions to build a foundation for a lasting relationship.
5. Attract “Churned/Low Value” Fans:
  • Implement price reductions strategically to re-attract these fans without compromising profit margins.
  • Use targeted promotions during sales events to encourage purchases.
  • Focus less marketing resources here compared to higher potential segments.
6. Optimize Marketing Resource Allocation:
  • Prioritize segments with high potential for loyalty and spending, such as Golden Fans and Promising Fans.
  • Allocate fewer resources to segments with lower engagement potential, like Churned/Low Value Fans.
  • Consider the different purchase behaviors within segments to fine-tune marketing strategies.
7. Adopt a Weighted RFM Model over a regular RFM Model:
  • Use a weighted RFM approach to better prioritize fan segments based on the club’s objectives.
  • Evaluate if the Monetary and Frequency are more critical than Recency in fan valuation, or if there is another order in feature importance. AHP can be used to research this.
8. Improve Classification Strategies:
  • Address challenges in classifying middle boundary clusters with targeted strategies.
  • Enhance the accuracy of fan segmentation for more precise marketing efforts.
9. Extend Methodology Beyond Football:
  • Adapt the methodology to other industries while considering different purchase dynamics.
  • Leverage the methodology’s strengths in segmentation to gain insights in varied contexts.

These recommendations aim to enhance fan engagement, increase merchandise revenue, and optimize marketing efforts based on the distinct behaviors and characteristics of each fan segment.

## 6 Discussions

This study has shown that the weighted RFM method may be a suitable approach for clustering Ajax football fans based on their merchandising behavior, where the proposed WRFM method appears to perform relatively better than the non-weighted RFM alternative. Moreover, this study also showed that the proposed methodology may provide reasonable results when used to cluster customers in a case outside the football industry. The findings of this study may assist AFC Ajax in closing the currently existing business gap, by implementing the discussed actionable insights which were derived from the established clusters. This in order to more effectively and efficiently utilize the club resources and advance in increasing the profitability among football fans. Marketing efforts now also may be targeted in accordance with the customer value of a fan, to maximize the return on investment and eventually achieve the defined goals in the multi-annual strategic programme of the club. Altogether, the findings of this study provide a great start for the club to implement data-driven decision making to enhance its marketing strategies and optimize the match between the club's supply and fan's demand, to eventually strengthen its competitive position in the football environment. The contribution of this study from the academic point of view involves the implementation and application of clustering techniques for customer segmentation within the football industry. The aim is to positively contribute to the current research gap of the field. This study's methodology may be reproduced in equivalent cases in the football or sports industry. The findings of this study may then serve as a benchmark, baseline for model appropriation, or comparative analysis to similar (future) studies in the field. One important limitation of this study includes that the described findings are not representative nor generalizable for other sports, or even other football clubs in The Netherlands or anywhere else in the world, as the developed model is specifically based on Ajax data, which may not be representative for any other population. The findings are also not representative for other departments of AFC Ajax, such as ticketing purchase behavior, business-to-business activities, or even offline business-to-customer merchandising behavior, as the developed models are limited to only the online business-to-customer merchandising of a fan, which potentially may have different behavioral dynamics than the other environments. Another limitation includes that only eight participants were involved in the AHP, which may reflect an unreliable and biased representation of the population for determining the relative importance of the weights. A minimum of 30 participants would have been a more suitable alternative, but unfortunately was not feasible due to time constraints.

A non-exhaustive list of limitation considerations includes: (1) Lack of a similar (W)RFM case in the football industry for proper model appropriation. (2) Although the proposed model showed a more suitable ranking, the difference between clusters 4 and 5 is relatively small, which may be debatable. (3) Fans that rarely purchase online but mainly offline, may be disadvantaged in the current setup. (4) Potentially a better classifier could have been developed by using hyperparameter optimization or another classification algorithm to further improve the performance. (5) Different findings could have been derived if AHP was taken at another moment of time, as the findings are a snapshot. Or even if another method than AHP was used for determining the weights. (6) And the effect of COVID-19 has to be taken into consideration regarding a fan's purchase behavior.

Another limitation in this study is its focus on a single entity or club, which restricts the generalizability of results. However, in the context of football clubs within the same geographical area, the revenue structure and fan engagement patterns often exhibit significant similarities. These similarities stem from factors such as regional economic conditions, cultural influences, and competitive dynamics, which standardize the revenue streams across clubs in the same area. For instance, clubs in a particular city or league typically share similar ticket pricing strategies, merchandising opportunities, and sponsorship deals due to comparable market conditions and fan demographics. For instance, Aslantaş et al. (48) demonstrated the effective segmentation of customers using K-means clustering and RFM metrics in the home appliance sector, which could be adapted to segment football fans based on their purchasing behaviors of tickets, merchandise, and memberships.

Despite these similarities, there are unique aspects of each club that must be considered when generalizing these models. Differences in club size, fan base, and historical success can impact revenue generation and fan behavior. Therefore, while the general framework of RFM and clustering algorithms can be applied broadly, it is essential to tailor the specific parameters and weighting of the models to account for club-specific nuances. The generalization of customer segmentation and CLV models to football and other sports is feasible and beneficial, leveraging methodologies that have proven effective in retail and other sectors. While the studies reviewed are limited by their focus on single entities, the consistent revenue patterns within the Ajax football club in the same geographical area suggest that these models can be adapted with appropriate customization.

Considerations for future work include the application of the methodology in other areas of Ajax, such as ticketing or business relations, other Dutch or European football clubs, or even other sports such as basketball. Other (non-exhaustive) considerations for future work include: (1) Further analyze the clusters by taking other features in consideration such as age or ticketing sales, to better understand the differences among clusters. (2) Investigate how fans move between clusters over time. (3) Apply A/B testing to evaluate if targeting the defined clusters results in an improved performance of the marketing activities, such as reactivating Churned/Low Value fans into active fans or converting Promising fans into Golden Fans. (4) Further evaluate the AHP findings to investigate the differences/similarities in opinions of Ajax staff regarding the important elements of a fan in terms of RFM. (5) Derive decision rules from the classifier to better understand the workings and boundaries of the model. (6) And implement alternative RFM variants such as RFMTC, RFD, RFE, or RFM-I and evaluate if they can outperform the proposed WRFM model. Finally, we will explore the application of these models across multiple clubs to validate their effectiveness and refine the parameters for broader sports contexts. This approach will enable football clubs and other sports entities to enhance fan engagement, optimize revenue streams, and develop targeted marketing strategies.

## Data Availability

The raw data in this article are not readily available due to confidential company data. Requests to access the datasets should be directed to the corresponding author.
