# Supplementary material for: Unlocking high-value football fans: unsupervised machine learning for customer segmentation and lifetime value
Source: Front Sports Act Living. 2024 Aug 22;6:1362489. doi: 10.3389/fspor.2024.1362489 (PMC11378343; doi:10.3389/fspor.2024.1362489)
Supplement: Supplementary file 1 [file Datasheet1.pdf]

Supplementary Materials of Research Article:
Unlocking high-value football fans: unsupervised machine learning for customer segmentation and lifetime value

Research Article Information
Authors: Karim Chouaten, Cristian Rodriguez Rivero, Frank Nack, Max Reckers
Frontiers in Sports and Active Living
Section Sports Management, Marketing, and Economics
doi: 10.3389/fspor.2024.1362489

This document contains all supplementary materials used in this research article. The figure and table numbers are in chronological order together with included materials in the research article.

Correspondence: Karim Chouaten

The supplementary figure and table materials are divided in the following sections:

- Section 1 - Methodology Flowchart
- Section 2 - Clustering Analysis Visualizations
- Section 3 - AHP Materials
- Section 4 - Characteristics of AFC Ajax Dataset
- Section 5 - Statistical Tests
- Section 6 - Confusion Matrix of the Decision Tree Classifier
- Section 7 - Results of Empirical Case

Please contact the corresponding author for any questions, remarks and/or requests.

SECTION 1 - METHODOLOGY FLOWCHART

Figure S1: Graphical representation of the steps of the proposed methodology of this study. Source: Created by Author himself based on the process flow of the Methodology section.

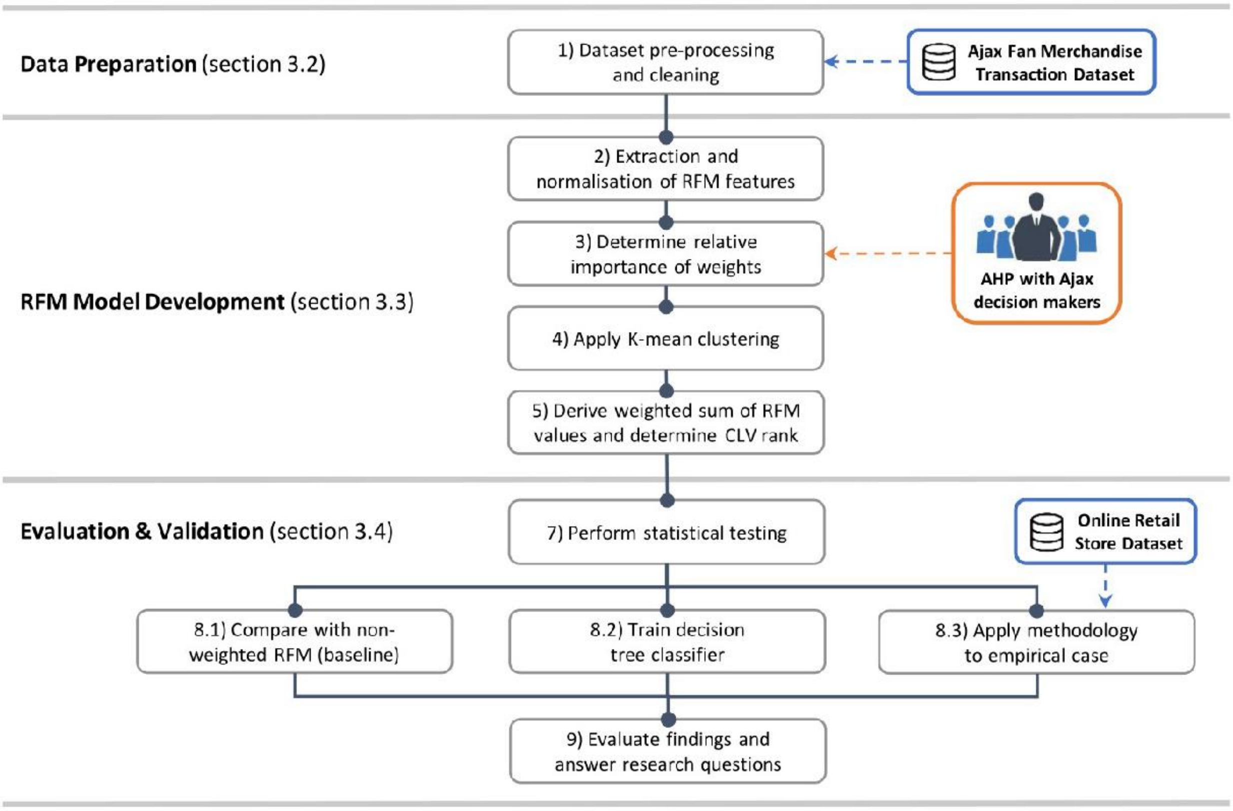

Note: sections 3.2, 3.3 and 3.4 refer to the sections in the research article.

SECTION 2 - CLUSTERING ANALYSIS VISUALIZATIONS

Figure S2: Scatterplot of the segments, where the x-axis includes the mean Recency on a reversed scale, y-axis the mean Monetary and the size the mean Frequency. Source: Author's own data analysis using dataset (49), refer to Methodology section for further details.

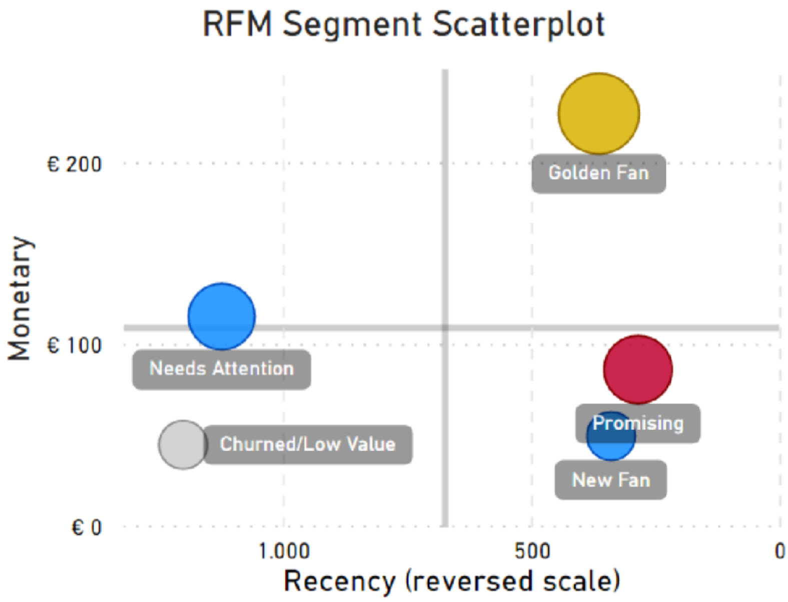

Figure S3: Shows a 3-dimensional scatterplot of the segments, where observations represent individual fans. The x-axis includes the fan's Recency, y-axis Monetary, and z-axis Frequency values. The color defines the segment type. The yellow pop-up shows the details of one observation in the *Golden Fan* area. Source: Author's own data analysis using dataset (49), refer to Methodology section for further details.

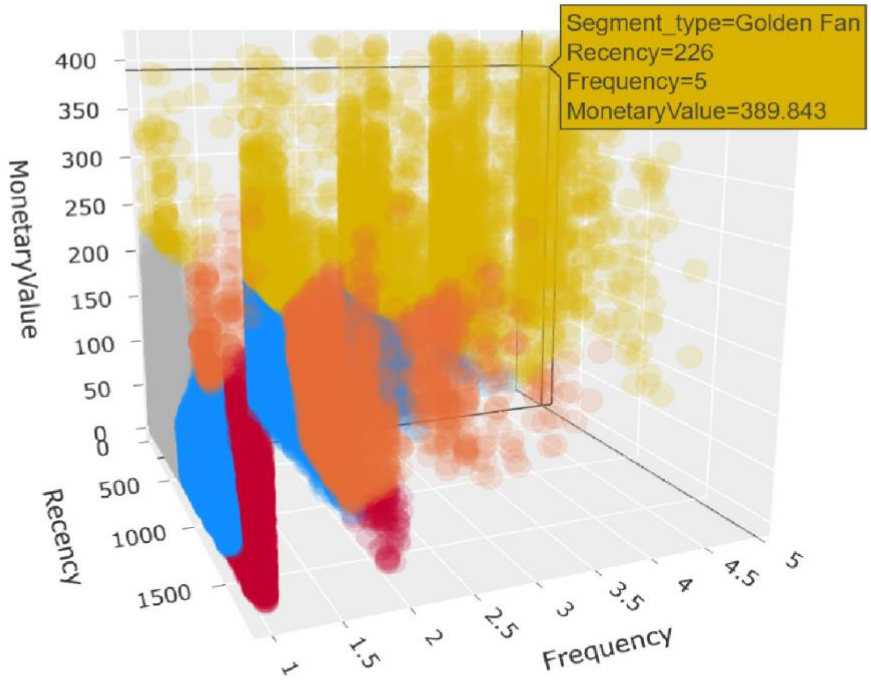

Figure S4: Pie chart showing the fan count distributions per segment in percentage. Source: Author's own data analysis using dataset (49), refer to Methodology section for further details.

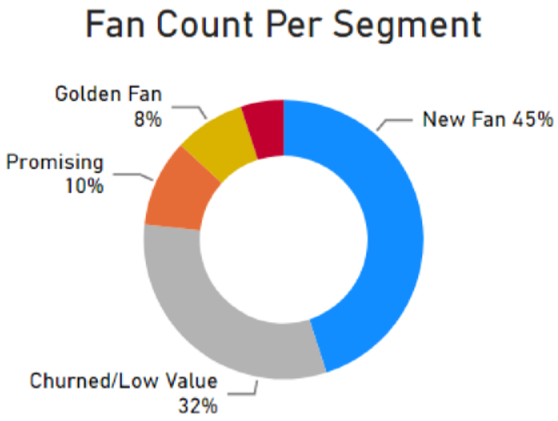

SECTION 3 - AHP MATERIALS

Table S1: AHP's fundamental scale of absolute numbers, used for the judgment of pairwise combinations. Source: Saaty (2003).

Scale	Description
1	Equal importance.
2	Intermediate between equal and weak.
3	Weak importance of one over another.
4	Intermediate between weak and strong.
5	Essential or strong importance
6	Intermediate between strong and demonstrated.
7	Demonstrated importance.
8	Intermediate between demonstrated and absolute.
9	Absolute or extreme importance.

Table S2: AHP results for the relative importance of the RFM weights, based on the input of eight domain experts within Ajax. The table shows the relative importance of each RFM variable along with the maximum consistency ratio of each participant's response. The group result summarizes the global importance and quantifies the RFM weights. Source: Author's own data analysis using the AHP survey results (refer to Methodology section for further details).

Participant	Recency	Frequency	Monetary	CRmax
**No. 1**	7.3%	72.7%	20.0%	1.0%
**No. 2**	10.5%	63.7%	25.8%	4.0%
**No. 3**	68.2%	23.6%	8.2%	0.2%
**No. 4**	9.4%	27.9%	62.7%	8.9%
**No. 5**	32.0%	12.2%	55.8%	1.9%
**No. 6**	61.5%	29.2%	9.3%	0.3%
**No. 7**	7.3%	20.0%	72.7%	1.0%
**No. 8**	18.8%	8.1%	73.1%	6.8%
**Group result**	**24.8%**	**34.3%**	**40.9%**	**0.1%**

SECTION 4 - CHARACTERISTICS AFC AJAX DATASET

Table S3: Summary statistics of the non-normalized RFM variables in the used Ajax online merchandising dataset. Source: Author's own data analysis using dataset (49), refer to Methodology section for further details.

	Recency	Frequency	Monetary
**Count**	315916	315916	315916
**Missing**	0	0	0
**Mean**	646.92	1.36	69.22
**Std**	486.25	0.78	64.73
**Min**	1.00	1.00	1.11
**25%**	272.00	1.00	21.06
**50%**	483.00	1.00	49.96
**75%**	993.00	1.00	94.92
**Max**	1828.00	5.00	418.02

Figure S5: Distribution plots shown for each normalised RFM variable of the Ajax online merchandising dataset. Source: Author's own data analysis using dataset (49), refer to Methodology section for further details.

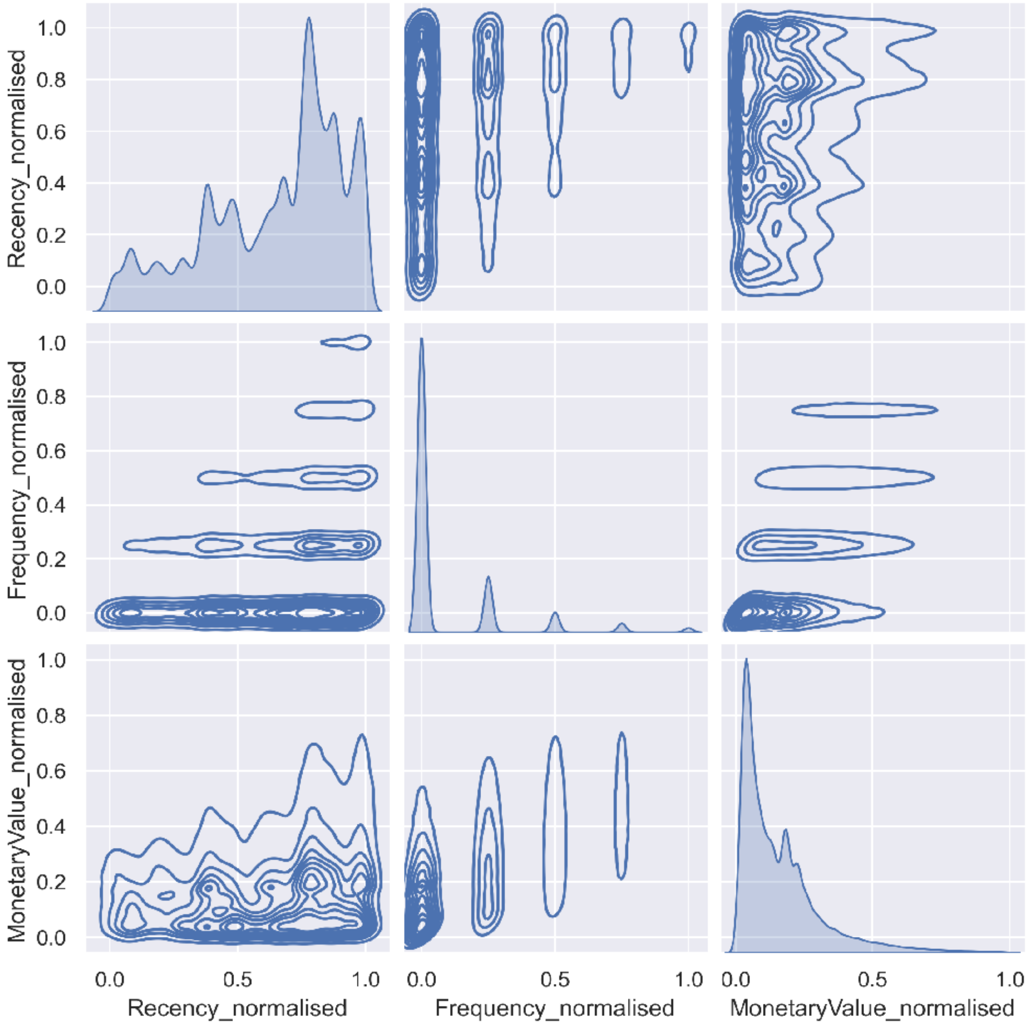

SECTION 5 - STATISTICAL TESTS

Table S4: Result of the Shapiro-Wilk Test for Normality. The p-value of each RFM variable is lower than the α of 0.05, thus the null-hypothesis for normality is rejected. Source: Author's own data analysis using dataset (49), refer to Methodology section for further details.

Shapiro-Wilk Test				
**Variable**	**W-Statistic**	**df**	**Sig.**	**Result**
Recency	0.925	315915	0	Reject
Frequency	0.528	315915	0	Reject
Monetary	0.805	315915	0	Reject

Table S5: Result of the Kruskall-Wallis H Test. Each p-value is lower than the stated α of 0.05, which means that the RFM values may significantly discriminate between the clusters. Source: Author's own data analysis using dataset (49), refer to Methodology section for further details.

Kruskall-Wallis H Test					
**Group**	**Variable**	**H-Statistic**	**df**	**Sig.**	**Result**
**WRFM Rank**	Recency	224932.9	7	0	Reject
	Frequency	299357.7	7	0	Reject
	Monetary	195431.2	7	0	Reject

Figure S6: Visual inspection for normality of each RFM variable using normal Q-Q plots. What may be concluded is that potentially none of the RFM variables may come from a normally distributed population. Source: Author's own data analysis using dataset (49), refer to Methodology section for further details.

(a) Recency variable

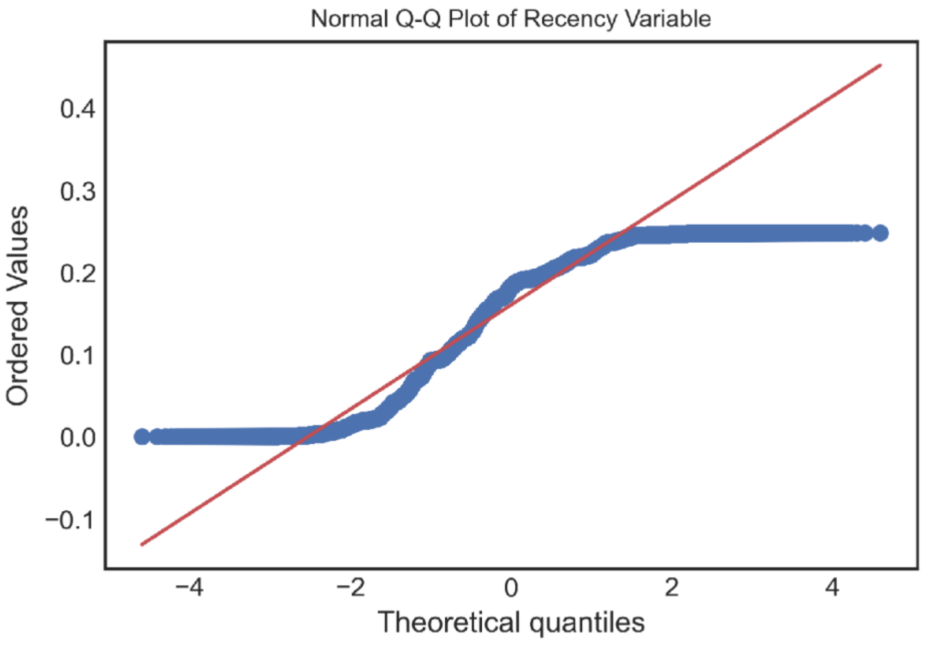

(b) Frequency variable

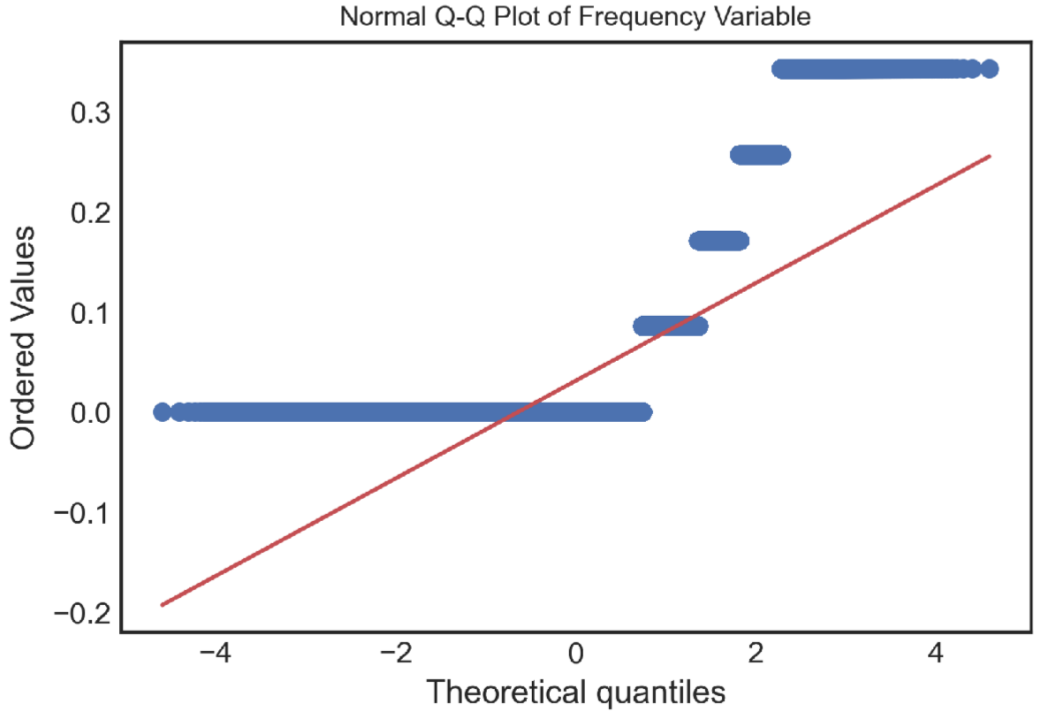

(c) Monetary variable

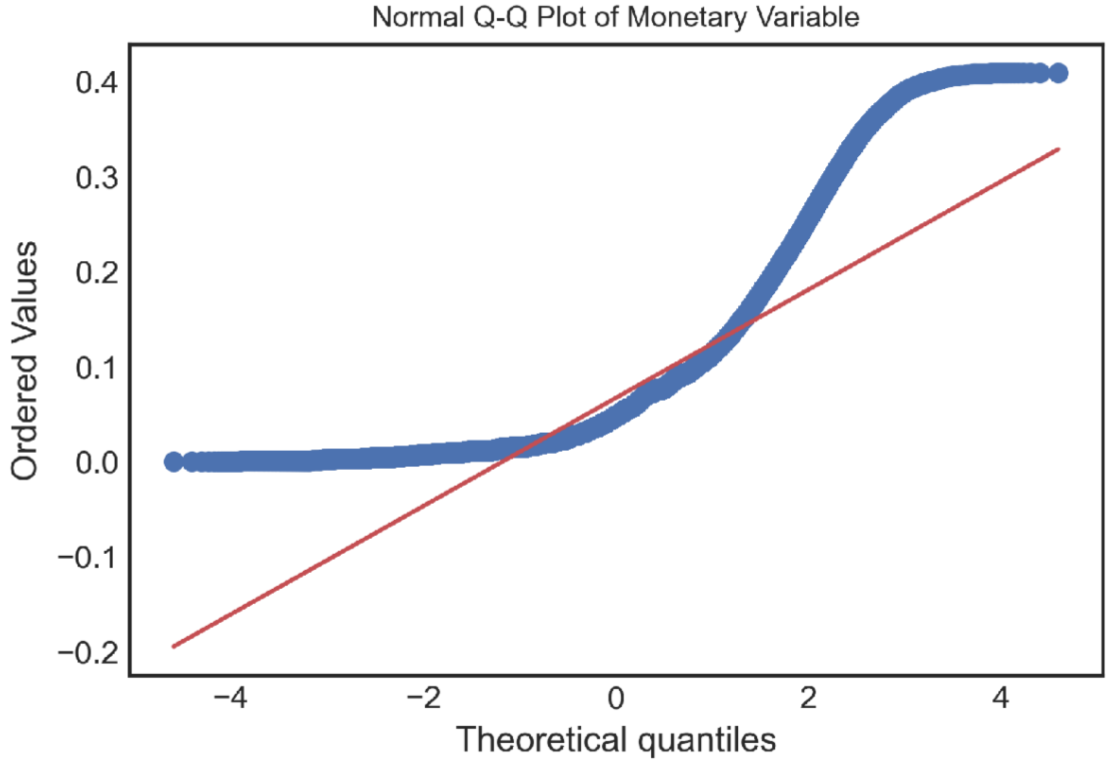

Table S6: Post-Hoc Dunn's Test applied on the Recency variable of each cluster. What can be concluded is that all ranks may be assumed significant, except for the ranks 2 versus 5. Source: Author's own data analysis using dataset (49), refer to Methodology section for further details.

Post-Hoc Dunn's Test - Recency Variable								
**WRFM Rank**	**1**	**2**	**3**	**4**	**5**	**6**	**7**	**8**
1	1.0	-	-	-	-	-	-	-
2	0.0	1.0	-	-	-	-	-	-
3	0.0	0.0	1.0	-	-	-	-	-
4	0.0	0.0	0.0	1.0	-	-	-	-
5	0.0	1.0*	0.0	0.0	1.0	-	-	-
6	0.0	0.0	0.0	0.0	0.0	1.0	-	-
7	0.0	0.0	0.0	0.0	0.0	0.0	1.0	-
8	0.0	0.0	0.0	0.0	0.0	0.0	0.0	1.0

Table S7: Post-Hoc Dunn's Test applied on the Frequency variable of each cluster. What can be concluded is that all ranks may be assumed significant, except for the ranks 5 versus 6, and ranks 7 versus 8. Source: Author's own data analysis using dataset (49), refer to Methodology section for further details.

Post-Hoc Dunn's Test - Frequency Variable								
**WRFM Rank**	**1**	**2**	**3**	**4**	**5**	**6**	**7**	**8**
1	1.0	-	-	-	-	-	-	-
2	0.0	1.0	-	-	-	-	-	-
3	0.0	0.0	1.0	-	-	-	-	-
4	0.0	0.0	0.0	1.0	-	-	-	-
5	0.0	0.0	0.0	0.0	1.0	-	-	-
6	0.0	0.0	0.0	0.0	1.0*	1.0	-	-
7	0.0	0.0	0.0	0.0	0.0	0.0	1.0	-
8	0.0	0.0	0.0	0.0	0.0	0.0	1.0*	1.0

Table S8: Post-Hoc Dunn's Test applied on the Monetary variable of each cluster. What can be concluded is that all ranks may be assumed to be significant. Source: Author's own data analysis using dataset (49), refer to Methodology section for further details.

Post-Hoc Dunn's Test - Monetary Variable								
**WRFM Rank**	**1**	**2**	**3**	**4**	**5**	**6**	**7**	**8**
1	1.0	-	-	-	-	-	-	-
2	0.0	1.0	-	-	-	-	-	-
3	0.0	0.0	1.0	-	-	-	-	-
4	0.0	0.0	0.0	1.0	-	-	-	-
5	0.0	0.0	0.0	0.0	1.0	-	-	-
6	0.0	0.0	0.0	0.0	0.0	1.0	-	-
7	0.0	0.0	0.0	0.0	0.0	0.0	1.0	-
8	0.0	0.0	0.0	0.0	0.0	0.0	0.0	1.0

SECTION 6 - CONFUSION MATRIX OF THE DECISION TREE CLASSIFIER

Table S9: Confusion matrix of the decision tree classifier trained on the Ajax dataset. Source: Author's own data analysis using dataset (49), refer to Methodology section for further details.

Predicted Rank \ Actual Rank	Rank 1	Rank 2	Rank 3	Rank 4	Rank 5	Rank 6	Rank 7	Rank 8	All
**Rank 1**	3014	6	0	165	0	0	0	0	3185
**Rank 2**	0	4341	223	93	13	0	22	0	4692
**Rank 3**	34	72	9169	242	0	0	46	0	9563
**Rank 4**	0	202	49	4058	0	0	318	20	4647
**Rank 5**	0	0	0	0	13925	79	126	0	14130
**Rank 6**	0	0	0	0	110	28501	84	0	28695
**Rank 7**	0	0	0	13	162	71	19033	128	19407
**Rank 8**	0	0	0	75	0	0	540	9841	10456
**All**	3048	4621	9441	4646	14210	28651	20169	9989	94775

SECTION 7 - RESULTS OF EMPIRICAL CASE

Figure S7: Distribution plots shown for each normalised RFM variable of the empirical Online Retail Store dataset. Source: Source: Author's own data analysis using dataset (46), refer to Methodology section for further details.

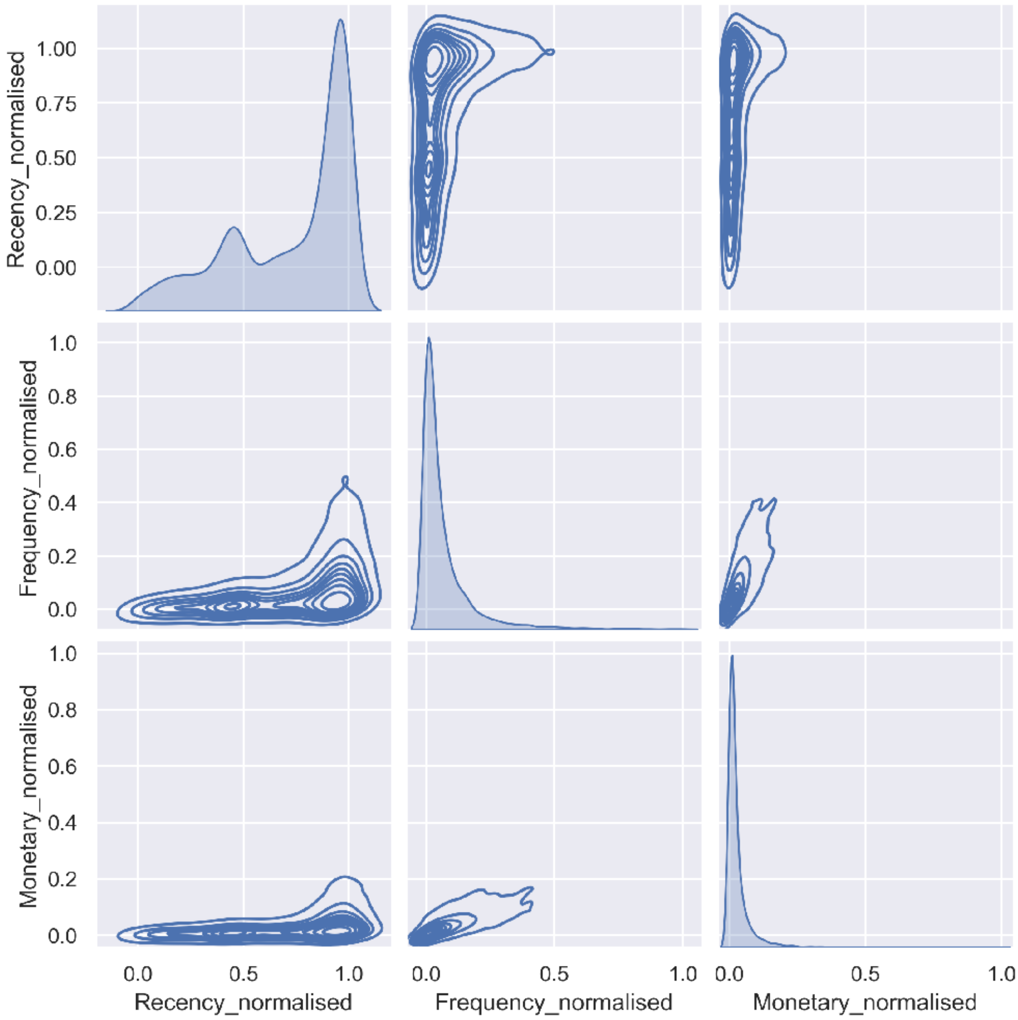

Table S10: Summary statistics of the non-normalized RFM variables in the empirical Online Retail Store dataset. Source: Author's own data analysis using dataset (46), refer to Methodology section for further details.

**Summary statistics of empirical dataset**			
	**Recency**	**Frequency**	**Monetary**
**Count**	5842	5842	5842
**Missing**	0	0	0
**Mean**	202.95	5.59	2270.02
**Std**	209.45	7.32	4549.53
**Min**	1.00	1.00	2.95
**25%**	27.00	1.00	346.32
**50%**	97.00	3.00	885.51
**75%**	381.00	7.00	2249.96
**Max**	739.00	70.00	74163.00

Table S11: Overview of the empirical model's clustering results. For each cluster the average non-weighted and nonnormalized R, F and M values are shown. Additionally, the pattern has been determined, by evaluating each RFM value if it is above or below the total mean. Based on the pattern, a segment name has been assigned to each cluster. The rank of the weighted RFM is also shown. The light cells with a star (*) mark the differences between the proposed and empirical models. Source: Author's own data analysis using dataset (46), refer to Methodology section for further details.

Clustering Results of Empirical Case							
**Cluster No.**	**Segment**	**# Fans**	**R (days)**	**F**	**M (Euro)**	**Pattern**	**WRFM Rank**
**6**	**Golden Customer**	48	32.25	50.15	23845.09	R↓ F↑ M↑	1
**3**	**Golden Customer**	176	25.84	29.10	13026.38	R↓ F↑ M↑	2
**7**	**Golden Customer**	459	34.78	15.63	6895.34	R↓ F↑ M↑	3*
**0**	**New Customer**	879	45.90	8.18	3187.79	R↓ F↓ M↓	4*
**4**	**New Customer**	1702	48.28	2.71	981.40	R↓ F↓ M↓	5
**5**	**Churned / Low Value**	879	229.28	3.04	1093.32	R↑ F↓ M↓	6*
**1**	**Churned / Low Value**	1072	417.56	2.42	802.14	R↑ F↓ M↓	7
**2**	**Churned / Low Value**	630	624.15	1.48	580.84	R↑ F↓ M↓	8
**Total average**	**-**	731	182.26	14.09	6301.56	-	-

Table S12: Performance results of the empirical decision tree classifier. For each cluster, the (in)correctly predicted clusters are shown, along with the accuracy rate (%), precision, recall and F1-score. The overall performance is summarized at the bottom, specified as overall (in)correctly predicted clusters, accuracy rate (%) and macro/weighted averages. Source: Author's own data analysis using dataset (46), refer to Methodology section for further details.

Decision Tree Classifier Results of Empirical Case (N = 1754)					
**WRFM Rank**	**Accuracy/error**	**Accuracy rate (%)**	**Precision**	**Recall**	**F1-score**
**1**	20/0	100.00%	1.00	0.95	0.98
**2**	54/1	98.18%	0.98	0.98	0.98
**3**	145/2	98.64%	0.99	1.00	0.99
**4**	245/2	99.19%	0.99	0.98	0.99
**5**	491/0	100.00%	1.00	1.00	1.00
**6**	273/6	97.85%	0.98	0.99	0.98
**7**	322/3	99.08%	0.99	0.99	0.99
**8**	188/2	98.95%	0.99	0.99	0.99
**Total:**	1738/16	**Macro average:**	0.99	0.99	0.99
**Accuracy (%):**	99.09%	**Weighted average:**	0.99	0.99	0.99
